# A Bioinformatics Analysis Reveals Novel Pathogens as Molecular Mimicry Triggers of Systemic Sclerosis

**DOI:** 10.31138/mjr.31.1.50

**Published:** 2020-03-31

**Authors:** Athanasios Gkoutzourelas, Maria Barmakoudi, Dimitrios P. Bogdanos

**Affiliations:** Department of Rheumatology and Clinical Immunology, Faculty of Medicine, University of Thessaly, Larissa, Greece

**Keywords:** autoantibody, autoimmunity, autoimmune rheumatic diseases, infection, molecular mimicry

## Abstract

A recent bioinformatic analysis revealing dominant B cell epitopes of systemic sclerosis-specific autoantibodies, including anti-centromere B, anti-topoisomerase I and anti-fibrillarin, has demonstrated the existence of several in silico antigenic mimics of pathogens that could act as triggers of the respective dominant autoepitopes. Based on those findings, the aim of the present study was to use a more comprehensive bioinformatic analysis. We demonstrated the presence of a plethora of novel microbial mimics, unnoticed by the studies so far conducted, which share remarkable amino acid similarities with the respective autoantigenic epitopes. This bioinformatic approach coupled by in vitro testing of the homologous self/non-self-mimics in serum samples from patients with systemic sclerosis may provide novel evidence of immunological cross-reactivity, implicating currently ignored or overlooked pathogens, which may indeed play a role in the induction of SSc-specific autoantibodies and assist efforts to understand the pathogenesis of this enigmatic disease.

## INTRODUCTION

Molecular mimicry has been proposed as a pathogenic mechanism for an autoimmune disease’s development, as well as a probe useful in uncovering its aetiologic agents.^1,2^ The concept of molecular mimicry is based on the abundant epidemiological, clinical, and experimental evidence of an association of infectious agents with specific autoimmune diseases, and observed immunological cross-reactivities between self-targets and microbial determinants.^3,4^ Immunological cross-reactivity is demonstrated when antibodies against a viral or microbial epitope (linear or conserved) can recognize a self-sequence of a known autoantigen, because of their antigenic similarity. In the case of linear epitopes, this similarity is likely to be a consequence of amino acid (aa) similarities between the core epitopic region of the respective self/non-self-epitopes.^2,4^ If this occurs, a cross-reactive immune response against the determinant shared by the host and the virus can evoke a tissue-specific immune response that is presumably capable of eliciting cell and tissue destruction. The probable mechanism is generation of autoantibodies or cytotoxic cross-reactive effector lymphocytes, that recognise specific determinants on target cells.^4^ By a complementary mechanism, the microbe can induce cellular injury and release self-antigens, which generate immune responses that cross-react with additional but distinct self-antigens, a mechanism which we have termed “multiple hit” molecular mimicry. However, the ultimate documentation of this mechanism has been elusive in humans. Presently, proof for molecular mimicry relies on the availability of structural data of viral and self-peptides that elicit cross-reactive immune responses and pathological features of the disease in experimental animal models.^2,4,5^

The information derived from animal studies is of undoubted value, but experimental models of human disease suffer from severe limitations since they rarely reproduce the human condition faithfully.^6–8^ Thus, several of the mimics that have been able to induce experimental autoimmune disease do not ultimately relate to the human setting. Molecular mimicry studies conducted in human biomaterial are almost impossible to undertake because they necessitate access to biomaterial (sera, cells, tissue) before exposure to the pathogen; such material is rarely accessible for testing.^9^

Several studies have implicated molecular mimicry as a likely mechanism responsible for the induction of systemic sclerosis (SSc)-specific autoantibodies.^10–18^ Sera from SSc patients recognize a sequence originated from human cytomegalovirus (CMV) late protein UL94 which appears to be homologous to the novel antigen-2 (NAG-2), which is expressed in endothelial cells.^12^ Anti-UL94 antibodies from SSc patients not only bind to NAG-2 on endothelial cells, but also can provoke apoptosis.^14^ This is rather intriguing, because when anti-UL94 CMV antibodies are bound to fibroblasts, they obtain a profibrotic phenotype. Another similarity between UL70 protein shared with Topoisomerase I, a major autoantigen in SSc, has been reported as a likely triggering factor in this disease, but no evidence of cross-reactivity has so far been obtained.^10^ A recent paper by Gourh et al. has revisited this topic and investigated the relationships between disease-related auto-antibodies, their respective autoantigens/autoepitopes, and genetic *HLA* associations, which confer susceptibility to geographically/ethnic distinct SSc cohorts.^19^ Through their meticulous analysis, and the application of bioinformatics tools, these investigators recognized viral-obtained peptidyl sequences from the Mimiviridae and Phycodnaviridae families, which are highly homologous to their SSc-specific autoantigenic counterparts, and could theoretically act as inducers of the disease *via* immunological cross-reactivity and molecular mimicry.^19^ Going through their identified viral sequences, it became apparent to us that the number of reported mimics was relatively smaller than what we thought we could obtain. We therefore decided to comprehensively assess the extent of self/non-self-mimicry and to provide a complete list of likely mimics also using a bioinformatics approach.

## MATERIAL AND METHODS

Basic Local Alignment Search Tool Protein (BLASTp), BLASTp2 (National Center for Biotechnology Information, NCBI, National Library of Medicine, Bethesda, MD, USA) (https://blast.ncbi.nlm.nih.gov/Blast.cgi?PAGE=Proteins) and Proteininfo (Rockefeller University, New York, NY, USA) (http://prowl.rockefeller.edu/prowl/proteininfo.html) computer-assisted programmes were used for aa comparisons and identification of peptidyl sequences homologues to those used as reference sequences reported by Gourh et al. “RQRAVALYFIDKLAL”, from the immunodominant topoisomerase I epitope and “LQEAAEAFLVHLFED” from the centromere A (CENPA) epitope.^19^ The (BLASTp) is a sequence comparison algorithm, enables comparison of a protein or peptide sequence with a database of sequences and identifies similar and potentially homologous sequences. The comparison of sequences is based on substitution matrices (such as BLOSUM62 that is used by BLAST by default) that score alignments of evolutionarily divergent protein sequences. We performed our BLASTp analyses as per Gourh et al. with slight modifications. When necessary, confirmatory Proteininfo analyses were performed, as we previously described.^20–25^

The Ethics Committee of the University General Hospital of Larissa, Greece granted an approval for the completion of the study in the University of Thessaly.

Statistical analyses for the significance of the observed similarities were automatically obtained by the acquisition of aligned sequences via BLASTp search engines.

## RESULTS

**Figure 1.**
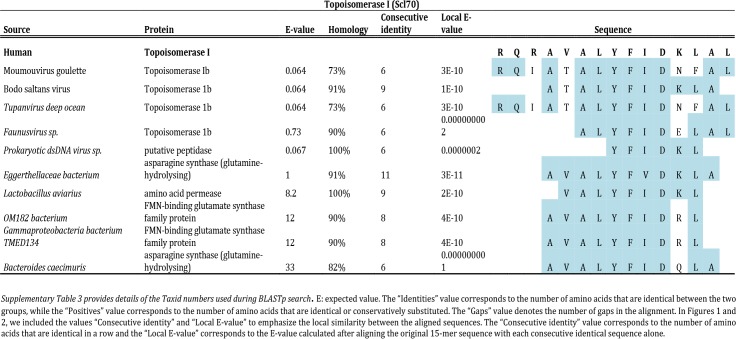
Representative microbial and viral mimics of topoisomerase I (Scleroderma 70, Scl70) dominant autoepitope. For each sequence (i.e. the 15-mer of and the 15-mer of topoisomerase I), the first five-homology pairs with viruses and the first five-homology pairs with bacteria were chosen, according to the following criteria: - The pairs ought to have at least 6 (≥6) consecutive homologous aa, allowing one aa difference, but of the same side chain group (i.e. I➔V or Y➔F) - Only one pair was chosen from same species - No pairs from phages were included - No pairs from uncultured bacteria or viruses were included - No hypothetical proteins were included

**Figure 2.**
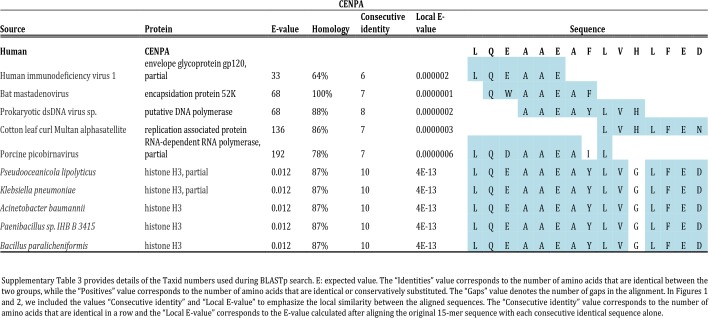
Representative microbial and viral mimics of centromere A (CENPA) dominant autoepitope. For each sequence (i.e. the 15-mer of and the 15-mer of CENPA), the first five-homology pairs with viruses and the first five-homology pairs with bacteria were chosen, according to the following criteria: - The pairs ought to have at least 6 (≥6) consecutive homologous aa, allowing one aa difference, but of the same side chain group (i.e. I➔V or Y➔F) - Only one pair was chosen from same species - No pairs from phages were included - No pairs from uncultured bacteria or viruses were included - No hypothetical proteins were included

**Figure 3.**
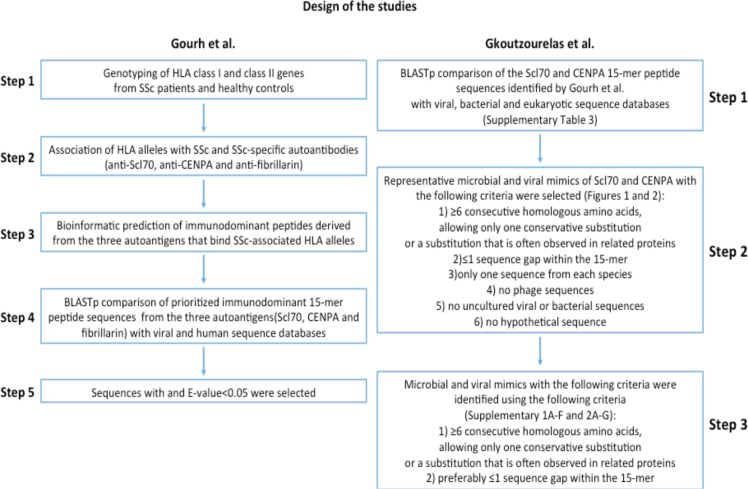
Step-wise approaches used by approaches used by Gourh et al.^19^ and the current study.

Taking advantage of the work performed by Gourh et al., who determined the immunodominant human topoisomerase I epitope, CENPA fibrillarin and its microbial mimics, we expanded this work emphasizing key elements that have gone unnoticed. First, we explored homology between the bioinformatically predicted immunodominant peptide sequences and microbial protein sequences to assess whether the mimicries we identified by BLASTp analysis differ to those reported by Gourh et al. (*Figure 1* and *2*, *Suppl. Tables 1–3*). In addition to that, we performed a Proteininfo analysis using 5meric sequences originated from topoisomerase I epitope “RQRAVALYFIDKLAL,” and CENPA sequence “LQEAAEAFLVHLFED”,^19^ and requested for identical motifs being present in microbial and human sequences. Initially, the bioinformatically predicted immunodominant peptide sequences from topoisomerase I were compared for homology with microbial protein sequence databases. The approaches used by Gourh et al. and by the present study are illustrated in *Figure 3*. The Several hundreds of homologous sequences were identified in various species some of which differed from those recently published.^19^ Gourh et al. reported only one sequence in topoisomerase I, “RQRAVALYFIDKLAL,” that had remarkable high-scored matches within the viral database at an Expected (E) value of <0.05. E-value represents the number of BLAST alignments with the observed score or higher than that are expected to occur by chance in a database search, and is a measure of the significance of homology. Some of the alignments produced by BLAST could be due to chance and not due to a biologically meaningful relationship between the two sequences. These alignments would have a high E-value. In contrast, alignments with low E-values are not random, and the two sequences might be related biologically. This is why Gourh et al. reported only high-scored matches with the E values of <0.05.

The Authors reported that the matched homologous peptides originated from the nucleocytoplasmic large DNA virus clade viruses, in the Mimiviridae family.^19^ We anticipated that analyses limited to matches with high quality matched, might lead to an underestimation of potential antigenic mimics for reasons we thoroughly discuss. An extensive list of matches provided by our analysis in *Suppl Tables 1* and *2*. All results were included, independently of their E-value, except hypothetical proteins with high E-value and results with big gaps.

No homologies found with human herpesviruses (including Epstein-Barr virus and human cytomegalovirus, which were blasted separately, as those viruses are very frequently involved in the induction of SSc-related autoimmunity), hepatitis C virus, *Brucella species* and *Staphylococci,* by selection.

Homologies with eukaryotes have not been included, as in their great majority have to deal with conserved DNA topoisomerases from fish, arthropoda, etc.

*Figures 1* and *2* depict representative homologies, which we considered of interest, as typical examples of self/non-self-similarities at the level of >5--mer aa length overlaps.^26^

## DISCUSSION

In the present study, we extended the understanding for the potential role played by molecular mimicry in the induction of SSc-specific autoimmunity. On the basis of a bioinformatics analysis, we provided an exhaustive list of microbial mimics of dominant B-cell autoantigenic epitopes, implying that several previously unnoticed pathogens may indeed play role in the development of autoreactive B cell responses related to SSc. This enormous number of pathogenic determinants shares remarkable aa homologies with two key autoepitopes, one originated from topoisomerase I (Scl70) and one from centromere A. Based on these findings, hypothesis-driven experiments working on the extent by which synthetic peptides spanning the respective aa sequences could be tested for antibody reactivity using sera from anti-centromere A and anti-Scl70 positive SSc patients. Such testing answers two key questions: first, how many of those peptides are targeted by cross-reactive antibodies? Ultimately, we could narrow down the list of biologically-meaningful peptides from few hundreds to a few dozens. Second, what is the relevance of the nature of the mimicking microbial peptides in relation to disease’s pathogenesis; ie, do these homologous peptides originate from pathogens which can infect humans, and to what extent?

The list of mimicking peptides is extensive, because it is well-known that homologies do not need to be remarkable in order to induce cross-reactivity; mimicries restricted to 5-6-aa long, may have great success in immunological terms.^26^ For example, Kanduc et al.26 found that 34 pentameric sequences from the viral capsid protein of human papillomavirus shared sequences with human proteins that are associated with cardiovascular diseases. Trost et al. showed that all human proteins present bacterial motifs at the level of 5-, 6-, 7- and 8-mer aa.^27,28^ Thus, aa similarity or even antigenic mimicry (shared motifs between foreign and self-antigens) is not sufficient to establish a significant connection. If the mimicries do not involve human proteins with biologic function such as enzymes or proteins with critical function, such targeting may embrace biologic meaningless, and must be disregarded. Foreign/human T-cell mimicry is more complicated, as motif sharing may involve very limited number of aa, and most molecular mimics cannot be identified by sequence alignment alone.^5^

Sharing of a 6-aa long peptide between hepatitis B virus DNA polymerase and myelin basic protein was enough to induce experimental encephalomyelitis in rabbits. In fact, databases currently exist, which provide open access to experimentally verified peptide sequences displaying molecular mimicry and immunological cross-reactivity, and can serve as sources of *in vivo* and *in vitro* research.^29^ For example, epidemiological studies, micro-biological data and immunological evidence of mimicry currently support a link between ankylosing spondylitis, HLA B27, and bacterial infection.^30^

A single substitution of one aa is more than enough to abrogate such an effect, highlighting the importance of the shared motifs. Dozens of papers have been published so far (including those generated by our group), reporting the existence of such mimicries that operate both at the B-cell (cross-reactive antibodies) and the T-cell level at various autoimmune diseases.^4,8,20–23,31,32^ We have also been able to show that mimicking alignments *per se* are not sufficient enough to reveal cross-reactive mimics.^24,25,33^ On the other hand, epitopic sequences, which do not share 100% identity, may well be able to act as cross-reactive targets of an antibody, if the dissimilar aa are not sufficient to change the antigenicity of the sort peptides. This is exemplified in particular at T-cell epitope mimics.^5^

Given the relatively limited number of previously observed similarities which involve SSc-specific autoantigens and pathogens, we hypothesized that BLAST search could identify more mimics, and therefore would uncover likely microbial and viral triggers of SSc autoantibodies. Such identification could provide a convincing theoretical explanation for non-self-triggered autoimmunity in SSc by the involvemnt of molecular (antigenic) mimicry and subsequent immunological cross-reaction. If the attack of the pathogen is met by the formation of strong T- and B-cells responses, aiming at controlling or preventing the infiltration of the virus in cells and tissues, and if the immune system is equipped with the ‘wrong’ genetic background, which restricts such responses at epitopic level that accidentally share homologies with self-epitopes, a cataclysm of reactions may happen which leads to autoimmunity and immune-mediated inflammation. Of particular interest was that we recognized several mimics that Gourh et al. did not identify, just because they could not fulfill their strict scoring criteria, which, by the way, are totally justifiable. With these results at hand, we speculate that local identical mimics must further be examined. Neither Gourh et al. nor we have experimentally tested the identified mimics. Immunological cross-reactivity can and must be defined through humoral and cellular studies involving synthetic peptides, which must be recognized by serum samples or T-cells from patients with SSc seropositive for their respective disease-specific autoantibodies. Only then, we can speculate on their potential pathogenic significance.

In conclusion, the present bioinformatics analysis allows the development of a hypothesis that is valid and can/must be tested *in vitro* and *in vivo*. It also provides important clues as to whether molecular mimicry is a likely mechanism for the induction of autoimmunity related to SSc, and opens a window of opportunity for specifically designed research focusing on the similarities so far published.

## Figures and Tables

**Suppl. Table 1A.** Topoisomerase I “RQRAVALYFIDKLAL,” BLASTp matches from viruses.

**Description**	**Score**	**E value**	**Identities**	**Positives**	**Gaps**
DNA topoisomerase IB [Hokovirus HKV1]	45.2 bits(99)	4.00E-06	14/15(93%)	14/15(93%)	0/15(0%)
DNA topoisomerase 1b [Megavirus vitis]	36.7 bits(79)	0.004	12/15(80%)	12/15(80%)	0/15(0%)
DNA topoisomerase 1b [Mimivirus sp. SH]	36.7 bits(79)	0.004	12/15(80%)	12/15(80%)	0/15(0%)
DNA topoisomerase 1b [Megavirus chiliensis]	36.7 bits(79)	0.004	12/15(80%)	12/15(80%)	0/15(0%)
hypothetical protein [Powai lake megavirus]	36.7 bits(79)	0.004	12/15(80%)	12/15(80%)	0/15(0%)
DNA topoisomerase IB [Klosneuvirus KNV1]	36.7 bits(79)	0.004	12/15(80%)	12/15(80%)	0/15(0%)
DNA topoisomerase IB [Catovirus CTV1]	35.0	0.016	12/15(80%)	12/15(80%)	0/15(0%)
DNA topoisomerase IB [Dasosvirus sp.]	34.6 bits(74)	0.022	12/15(80%)	12/15(80%)	0/15(0%)
DNA topoisomerase 1b [Edafosvirus sp.]	34.1 bits(73)	0.032	11/14(79%)	11/14(78%)	0/14(0%)
dna topoisomerase 1b [Tupanvirus soda lake]	33.3 bits(71)	0.063	11/15(73%)	11/15(73%)	0/15(0%)
topoisomerase 1b [Moumouvirus goulette]	33.3 bits	0.063	11/15(73%)	11/15(73%)	0/15(0%)
topoisomerase 1b [Bodo saltans virus]	33.3 bits(71)	0.063	10/11(91%)	10/11(90%)	0/11(0%)
topoisomerase 1b [Tupanvirus deep ocean]	33.3 bits(71)	0.063	11/15(73%)	11/15(73%)	0/15(0%)
topoisomerase 1b [Moumouvirus australiensis]	33.3 bits(71)	0.063	11/15(73%)	11/15(73%)	0/15(0%)
topoisomerase 1 [Moumouvirus maliensis]	33.3 bits(71)	0.063	11/15(73%)	11/15(73%)	0/15(0%)
DNA topoisomerase 1b [Acanthamoeba polyphaga moumouvirus]	33.3 bits(71)	0.063	11/15(73%)	11/15(73%)	0/15(0%)
DNA topoisomerase [Saudi moumouvirus]	33.3 bits(71)	0.063	11/15(73%)	11/15(73%)	0/15(0%)
DNA topoisomerase 1b [Moumouvirus Monve]	33.3 bits(71)	0.064	11/15(73%)	11/15(73%)	0/15(0%)
Topoisomerase 1b [Faunusvirus sp.]	30.3 bits(64)	0.72	9/10(90%)	9/10(90%)	0/10(0%)
putative HNH homing endonuclease [uncultured Caudovirales phage]	27.8 bits(58)	5.8	8/10(80%)	9/10(90%)	0/10(0%)
virion structural protein [Pseudomonas phage 201phi2-1]	26.5 bits(55)	17	7/7(100%)	7/7(100%)	0/7(0%)
putative 26S proteasome non-ATPase regulatory subunit 8-like protein	24.8 bits(51)	67	7/8(88%)	7/8(87%)	0/8(0%)
Serine/threonine protein kinase [Pandoravirus quercus]	24.4 bits(50)	95	7/7(100%)	7/7(100%)	0/7(0%)
ribonucleotide reductase of class III (anaerobic), large subunit [Proteus phage PM2]	24.4 bits(50)	95	7/8(88%)	7/8(87%)	0/8(0%)
anaerobic NTP reductase large subunit [Shigella phage vB_SdyM_006]	24.4 bits(50)	95	7/8(88%)	7/8(87%)	0/8(0%)
anaerobic NTP reductase large subunit [Proteus phage phiP4-3]	24.4 bits(50)	95	7/8(88%)	7/8(87%)	0/8(0%)
large tegument protein [Bovine gammaherpesvirus 6]	24.4 bits(50)	96	8/10(80%)	8/10(80%)	0/10(0%)
pol protein, partial [Human immunodeficiency virus 1]	24.0 bits(49)	133	7/9(78%)	7/9(77%)	0/9(0%)
Putative peptidase [Prokaryotic dsDNA virus sp.]	24.0 bits(49)	135	6/6(100%)	6/6(100%)	0/6(0%)
modification methylase [Prochlorococcus phage MED4-213]	24.0 bits(49)	135	7/10(70%)	8/10(80%)	0/10(0%)
RING finger domain protein [Faunusvirus sp.]	24.0 bits(49)	135	6/7(86%)	7/7(100%)	0/7(0%)
leucyl-tRNA synthetase [Hokovirus HKV1]	24.0 bits(49)	135	6/6(100%)	6/6(100%)	0/6(0%)
polyprotein [Porcine epidemic diarrhea virus]	24.0 bits(49)	136	6/8(75%)	7/8(87%)	0/8(0%)
polyprotein, partial [Hepatitis C virus subtype 1b]	23.5	190	6/8(75%)	8/8(100%)	0/8(0%)
minor tail protein [Microbacterium phage Zeta1847]	23.5 bits(48)	191	6/6(100%)	6/6(100%)	0/6(0%)

**Suppl. Table 1B.** Topoisomerase I “RQRAVALYFIDKLAL,” BLASTp matches from bacteria.

**Description**	**Score**	**E value**	**Identities**	**Positives**	**Gaps**
hypothetical protein [Ruminococcaceae bacterium]	36.7	0.26	12/15(80%)	12/15(80%)	0/15(0%)
asparagine synthase (glutamine-hydrolyzing) [Eggerthellaceae bacterium]	35.0	1.0	10/11(91%)	11/11(100%)	0/11(0%)
hypothetical protein A2622_08350 [Bdellovibrionales bacterium RIFCSPHIGHO2_01_FULL_40_29]	32.9	5.8	9/11(82%)	10/11(90%)	0/11(0%)
amino acid permease [Lactobacillus aviarius]	32.5	8.2	9/9(100%)	9/9(100%)	0/9(0%)
amino acid permease [Lactobacillus aviarius]	32.5	8.2	9/9(100%)	9/9(100%)	0/9(0%)
FMN-binding glutamate synthase family protein [OM182 bacterium]	32.0	12	9/10(90%)	9/10(90%)	0/10(0%)
FMN-binding glutamate synthase family protein [Gammaproteobacteria bacterium TMED134]	32.0	12	9/10(90%)	9/10(90%)	0/10(0%)
FMN-binding glutamate synthase family protein [Gammaproteobacteria bacterium]	32.0	12	9/10(90%)	9/10(90%)	0/10(0%)
efflux RND transporter permease subunit [Halothiobacillus neapolitanus]	30.8	33	10/15(67%)	11/15(73%)	0/15(0%)
MFS transporter [Halothiobacillus sp. 15-55-196]	30.8	33	10/15(67%)	11/15(73%)	0/15(0%)
asparagine synthase (glutamine-hydrolyzing) [Bacteroides caecimuris]	30.8	33	9/11(82%)	10/11(90%)	0/11(0%)
asparagine synthase (glutamine-hydrolyzing) [Cryptobacterium curtum]	30.8	33	9/11(82%)	10/11(90%)	0/11(0%)
asparagine synthase (glutamine-hydrolyzing) [Eubacterium ventriosum]	30.3	46	8/10(80%)	10/10(100%)	0/10(0%)
amino acid permease [Lactobacillus aviarius]	30.3	46	8/9(89%)	9/9(100%)	0/9(0%)
efflux RND transporter permease subunit [Halothiobacillus sp. LS2]	29.9	66	8/10(80%)	9/10(90%)	0/10(0%)
diguanylate cyclase/phosphodiesterase [Paucimonas lemoignei]	29.9	66	10/12(83%)	10/12(83%)	1/12(8%)
quinone oxidoreductase [Bradyrhizobium sp. INPA54B]	29.9	66	9/11(82%)	9/11(81%)	0/11(0%)
quinone oxidoreductase [bacterium RmlP001]	29.9	66	9/11(82%)	9/11(81%)	0/11(0%)
tyrosine recombinase [Polymorphobacter sp. DJ1R-1]	29.9	66	10/13(77%)	11/13(84%)	1/13(7%)
(d)CMP kinase [Cytophaga aurantiaca]	29.9	66	8/9(89%)	9/9(100%)	0/9(0%)
cytidylate kinase [Flammeovirgaceae bacterium]	29.9	66	8/10(80%)	9/10(90%)	0/10(0%)
HNH endonuclease [Salinisphaera japonica]	29.9	66	9/13(69%)	9/13(69%)	0/13(0%)
efflux RND transporter permease subunit [Rhizobiales bacterium]	29.5	93	8/10(80%)	10/10(100%)	0/10(0%)
lantibiotic dehydratase [Pedobacter yulinensis]	29.5	93	8/8(100%)	8/8(100%)	0/8(0%)
efflux RND transporter permease subunit [Thiohalospira halophila]	29.5	93	8/9(89%)	8/9(88%)	0/9(0%)
mechanosensitive ion channel [Mitsuaria sp. 7]	29.5	93	8/10(80%)	9/10(90%)	0/10(0%)
phosphoenolpyruvate synthase [Niabella ginsenosidivorans]	29.5	93	8/8(100%)	8/8(100%)	0/8(0%)
accessory Sec system translocase SecA2 [Paenibacillus sp. Leaf72]	29.5	93	10/12(83%)	10/12(83%)	1/12(8%)
ABC transporter permease [Desulfocapsa sp.]	29.5	93	8/8(100%)	8/8(100%)	0/8(0%)
RagB/SusD family nutrient uptake outer membrane protein [Pedobacter ginsengisoli]	29.5	93	8/8(100%)	8/8(100%)	0/8(0%)
acyl--CoA ligase [Proteiniclasticum ruminis]	29.5	93	8/8(100%)	8/8(100%)	0/8(0%)
RelA/SpoT domain-containing protein [Clostridiales bacterium SYSU GA17129]	29.5	93	8/8(100%)	8/8(100%)	0/8(0%)
DUF3696 domain-containing protein [Vulcanococcus limneticus]	29.5	93	8/9(89%)	9/9(100%)	0/9(0%)
ABC transporter permease [Candidatus Syntrophosphaera thermopropionivorans]	29.5	93	8/8(100%)	8/8(100%)	0/8(0%)
(d)CMP kinase [Fabibacter pacificus]	29.5	93	8/9(89%)	8/9(88%)	0/9(0%)
SufE family protein [Bacteroides salanitronis]	29.1	132	8/9(89%)	8/9(88%)	0/9(0%)
helix-turn-helix domain-containing protein [Bifidobacterium adolescentis]	29.1	132	8/9(89%)	8/9(88%)	0/9(0%)
(d)CMP kinase [Cytophagales bacterium B6]	29.1	132	8/8(100%)	8/8(100%)	0/8(0%)
cytidylate kinase [Candidatus Marinimicrobia bacterium]	29.1	132	8/8(100%)	8/8(100%)	0/8(0%)
Predicted hydrolase (HAD superfamily) [Pseudomonas aeruginosa]	25.2	28	8/11(73%)	9/11(81%)	2/11(18%)
methyltransferase domain-containing protein [Pseudomonas aeruginosa]	25.2	28	8/11(73%)	9/11(81%)	2/11(18%)
hypothetical protein ACS96_27930 [Pseudomonas aeruginosa]	24.4	56	7/7(100%)	7/7(100%)	0/7(0%)
TetR/AcrR family transcriptional regulator [Pseudomonas aeruginosa]	24.0	79	7/8(88%)	7/8(87%)	0/8(0%)

**Suppl. Table 1C.** Topoisomerase I “RQRAVALYFIDKLAL,” BLASTp matches from Gram+ bacteria in particular.

**Description**	**Score**	**E value**	**Identities**	**Positives**	**Gaps**
hypothetical protein [Ruminococcaceae bacterium]	36.7 bits(79)	0.041	12/15(80%)	12/15(80%)	0/15(0%)
amino acid permease [Lactobacillus aviarius]	2.5	1.3	9/9(100%)	9/9(100%)	0/9(0%)
asparagine synthase (glutamine-hydrolyzing) [Eubacterium ventriosum]	30.3	7.4	8/10(80%)	10/10(100%)	0/10(0%)
accessory Sec system translocase SecA2 [Paenibacillus sp. Leaf72]	29.5 bits(62)	15	10/12(83%)	10/12(83%)	1/12(8%)
acyl--CoA ligase [Proteiniclasticum ruminis]	9.5	15	8/8(100%)	8/8(100%)	0/8(0%)
RelA/SpoT domain-containing protein [Clostridiales bacterium SYSU GA17129]	29.5 bits(62)	15	8/8(100%)	8/8(100%)	0/8(0%)
hydrolase [Clostridium sp. N3C]	29.5 bits(62)	15	8/9(89%)	9/9(100%)	0/9(0%
(d)CMP kinase [Anaerocolumna aminovalerica]	29.1 bits(61)	21	8/8(100%)	8/8(100%)	0/8(0%)
WxPxxD family membrane protein [Falsibacillus sp. GY 10110]	29.1 bits(61)	21	8/9(89%)	8/9(88%)	0/9(0%)
PucR family transcriptional regulator [Hydrogenibacillus schlegelii]	28.6 bits(60)	30	8/10(80%)	9/10(90%)	0/10(0%)
endonuclease [Staphylococcus pettenkoferi]	27.8 bits(58)	59	8/10(80%)	9/10(90%)	0/10(0%)
cytidylate kinase [Coprococcus sp. CAG:782]	27.8 bits(58)	60	7/9(78%)	9/9(100%)	0/9(0%)
(d)CMP kinase [Coprococcus sp. OM04-5BH]	27.8 bits(58)	60	7/9(78%)	9/9(100%)	0/9(0%)
(d)CMP kinase [Lachnospiraceae bacterium]	27.8 bits(58)	60	7/9(78%)	9/9(100%)	0/9(0%)
N-acetylneuraminate lyase [Streptococcus pneumoniae]	27.8 bits(58)	60	9/12(75%)	9/12(75%)	0/12(0%)
ABC transporter permease subunit [Bacillus psychrosaccharolyticus]	27.8 bits(58)	60	8/9(89%)	8/9(88%)	0/9(0%)
DegV family protein [Paenibacillus wynnii]	27.8 bits(58)	60	7/10(70%)	9/10(90%)	0/10(0%)
DUF4097 domain-containing protein [Bacillus massilionigeriensis]	27.8 bits(58)	60	9/12(75%)	10/12(83%)	0/12(0%)
glycogen synthase GlgA [Desulfosporosinus sp. OL]	27.8 bits(58)	60	9/12(75%)	10/12(83%)	0/12(0%)
cupin domain-containing protein [Lysinibacillus varians]	27.4 bits(57)	84	8/10(80%)	9/10(90%)	0/10(0%)

**Suppl. Table 1D.** Topoisomerase I “RQRAVALYFIDKLAL,” BLASTp matches from *Enterococci* in particular.

**Description**	**Score**	**E value**	**Identities**	**Positives**	**Gaps**
hypothetical protein [Enterococcus raffinosus]	26.5 bits(55)	5.7	7/7(100%)	7/7(100%)	0/7(0%)
GNAT family N-acetyltransferase [Enterococcus mediterraneensis]	25.2 bits(52)	16	7/7(100%)	7/7(100%)	0/7(0%)
bifunctional glutamate--cysteine ligase GshA/glutathione synthetase GshB [Enterococcus saccharolyticus]	25.2 bits(52)	16	7/7(100%)	7/7(100%)	0/7(0%)
AAA family ATPase [Enterococcus avium]	24.4 bits(50)	33	6/7(86%)	7/7(100%)	0/7(0%)

**Suppl. Table 1E.** Topoisomerase I “RQRAVALYFIDKLAL,” BLASTp matches from Streptococci in particular.

**Description**	**Score**	**E value**	**Identities**	**Positives**	**Gaps**
N-acetylneuraminate lyase [Streptococcus pneumoniae]	27.8	6.5	9/12(75%)	9/12(75%)	0/12(0%)
hypothetical protein [Streptococcus pneumoniae]	27.4	9.1	9/13(69%)	9/13(69%)	2/13(15%)
GNAT family N-acetyltransferase [Streptococcus suis]	27.4	9.2	9/12(75%)	10/12(83%)	2/12(16%)
hypothetical protein SAMN04487837_0195 [Streptococcus equinus]	26.5	18	7/7(100%)	7/7(100%)	0/7(0%)
aminoacyl-tRNA deacylase [Streptococcus mitis]	25.2	53	7/7(100%)	7/7(100%)	0/7(0%)
MULTISPECIES: IS3 family transposase [Actinobacteria]	24.8	74	7/8(88%)	7/8(87%)	0/8(0%)

**Suppl. Table 1F.** Topoisomerase I “RQRAVALYFIDKLAL,” BLASTp matches from *Helicobacter* in particular.

**Description**	**Score**	**E value**	**Identities**	**Positives**	**Gaps**
ABC-F family ATP-binding cassette domain-containing protein [Helicobacter canis]	26.5	9.3	7/7(100%)	7/7(100%)	0/7(0%)
LptF/LptG family permease [Helicobacter japonicus]	25.7	19	7/7(100%)	7/7(100%)	0/7(0%)
tRNA (guanosine(46)-N7)-methyltransferase TrmB [Helicobacter pylori]	24.8	38	9/12(75%)	9/12(75%)	2/12(16%)

**Suppl. Table 2A.** CENPA sequence “LQEAAEAFLVHLFED” BLASTp matches from bacteria.

**Description**	**Score**	**E value**	**Identities**	**Positives**	**Gaps**
hypothetical protein, partial [Macrococcus caseolyticus]	41.8	0.004	13/15(87%)	14/15(93%)	0/15(0%)
hypothetical protein D6861_11520 [Macrococcus caseolyticus]	41.8	0.004	13/15(87%)	14/15(93%)	0/15(0%)
hypothetical protein, partial [Brucella melitensis]	41.8	0.004	13/15(87%)	14/15(93%)	0/15(0%)
hypothetical protein, partial [Homoserinimonas sp. OAct 916]	40.5	0.011	13/15(87%)	14/15(93%)	0/15(0%)
histone H3, partial [Pseudooceanicola lipolyticus]	40.5	0.011	13/15(87%)	14/15(93%)	0/15(0%
hypothetical protein CVM52_26375 [Pseudooceanicola lipolyticus]	40.5	0.011	13/15(87% )	14/15(93%)	0/15(0%)
hypothetical protein DKP78_14540, partial [Enterococcus faecium]	40.5	0.011	13/15(87%)	14/15(93%)	0/15(0%)
histone H3, partial [Klebsiella pneumoniae]	40.5	0.011	13/15(87%)	14/15(93%)	0/15(0%)
histone H3, partial [Soehngenia saccharolytica]	40.5	0.011	13/15(87%)	14/15(93%)	0/15(0%)
hypothetical protein, partial [Escherichia coli]	40.5	0.011	13/15(87%)	14/15(93%)	0/15(0%)
histone H3, partial [Klebsiella pneumoniae]	40.5	0.011	13/15(87%)	14/15(93%)	0/15(0%)
hypothetical protein, partial [Escherichia coli]	40.5	0.011	13/15(87%)	14/15(93%)	0/15(0%)
histone H3 [Acinetobacter baumannii]	40.5	0.011	13/15(87%)	14/15(93%)	0/15(0%)
histone H3, partial [Klebsiella pneumoniae]	40.5	0.011	13/15(87%)	14/15(93%)	0/15(0%)
hypothetical protein, partial [Nitriliruptoraceae bacterium ZYF776]	40.5	0.011	13/15(87%)	14/15(93%)	0/15(0%)
histone H3 [Paenibacillus sp. IHB B 3415]	40.5	0.011	13/15(87%)	14/15(93%)	0/15(0%)
histone H3 [Bacillus paralicheniformis]	40.5	0.011	13/15(87%)	14/15(93%)	0/15(0%)
histone H3 [Acinetobacter baumannii]	40.5	0.011	13/15(87%)	14/15(93%)	0/15(0%)
hypothetical protein DRB13_30320 [Klebsiella pneumoniae]	40.5	0.011	13/15(87%)	14/15(93%)	0/15(0%)
hypothetical protein, partial [Escherichia coli]	40.5	0.012	13/15(87%)	14/15(93%)	0/15(0%)
hypothetical protein, partial [Escherichia coli]	40.5	0.012	13/15(87%)	14/15(93%)	0/15(0%)
hypothetical protein [Acinetobacter baumannii]	40.5	0.012	13/15(87%)	14/15(93%)	0/15(0%)
histone H3, partial [Arthrobacter sp. SX1312]	40.5	0.012	13/15(87%)	14/15(93%)	0/15(0%)
hypothetical protein, partial [Acinetobacter baumannii]	40.5	0.012	13/15(87%)	14/15(93%)	0/15(0%)
hypothetical protein, partial [Acinetobacter baumannii]	40.5	0.012	13/15(87%)	14/15(93%)	0/15(0%)
hypothetical protein [Acinetobacter baumannii]	40.5	0.012	13/15(87%)	14/15(93%)	0/15(0%)
hypothetical protein, partial [Acinetobacter baumannii]	40.5	0.012	13/15(87%)	14/15(93%)	0/15(0%)
histone H3 [Paenibacillus azotifigens]	38.8	0.046	12/15(80%)	13/15(86%)	0/15(0%)
histone H3 [Anaplasma phagocytophilum]	38.0	0.090	12/15(80%)	13/15(86%)	0/15(0%)
hypothetical protein [Anaplasma phagocytophilum]	38.0	0.090	12/15(80%)	13/15(86%)	0/15(0%)
hypothetical protein [Synechococcus sp. CPC35]	38.0	0.091	12/15(80%)	13/15(86%)	0/15(0%)
histone H3 [Anaplasma phagocytophilum]	38.0	0.091	12/15(80%)	13/15(86%)	0/15(0%)
hypothetical protein, partial [Nitriliruptoraceae bacterium ZYF776]	38.0	0.091	12/15(80%)	13/15(86%)	0/15(0%)
hypothetical protein [Actinobacteria bacterium]	38.0	0.091	12/15(80%)	13/15(86%)	0/15(0%)
histone H3 [Acinetobacter baumannii]	38.0	0.091	12/15(80%)	13/15(86%)	0/15(0%)
histone H3, partial [Pseudomonas aeruginosa]	38.0	0.091	12/15(80%)	13/15(86%)	0/15(0%)
hypothetical protein [Alteromonas sp.]	38.0	0.091	12/15(80%)	13/15(86%)	0/15(0%)
histone H3 [Salmonella enterica]	38.0	0.091	12/15(80%)	13/15(86%)	0/15(0%)
histone H3, partial [Vibrio parahaemolyticus]	38.0	0.091	12/15(80%)	13/15(86%)	0/15(0%)
histone H3, partial [Pseudomonas aeruginosa]	38.0	0.091	12/15(80%)	13/15(86%)	0/15(0%)
histone H3, partial [Acinetobacter baumannii]	38.0	0.092	12/15(80%)	13/15(86%)	0/15(0%)
histone H3, partial [Pseudomonas aeruginosa]	38.0	0.092	12/15(80%)	13/15(86%)	0/15(0%)
hypothetical protein, partial [Klebsiella pneumoniae]	38.0	0.092	12/15(80%)	13/15(86%)	0/15(0%)
hypothetical protein, partial [Acinetobacter baumannii]	38.0	0.092	12/15(80%)	13/15(86%)	0/15(0%)
hypothetical protein, partial [Pseudomonas aeruginosa]	38.0	0.092	12/15(80%)	13/15(86%)	0/15(0%)
hypothetical protein, partial [Acinetobacter baumannii]	38.0	0.092	12/15(80%)	13/15(86%)	0/15(0%)
hypothetical protein, partial [Acinetobacter baumannii]	38.0	0.092	12/15(80%)	13/15(86%)	0/15(0%)
hypothetical protein, partial [Nitriliruptoraceae bacterium ZYF776]	38.0	0.092	12/15(80%)	13/15(86%)	0/15(0%)
hypothetical protein, partial [Acinetobacter baumannii]	38.0	0.092	12/15(80%)	13/15(86%)	0/15(0%)
hypothetical protein E8P77_31285, partial [Soehngenia saccharolytica]	38.0	0.093	12/15(80%)	13/15(86%)	0/15(0%)
hypothetical protein, partial [Acinetobacter baumannii]	38.0	0.093	12/15(80%)	13/15(86%)	0/15(0%)
hypothetical protein, partial [Enterococcus faecium]	38.0	0.093	12/15(80%)	13/15(86%)	0/15(0%)
hypothetical protein, partial [Enterobacter cloacae complex sp. 2DZ2F20B]	38.0	0.093	12/15(80%)	13/15(86%)	0/15(0%)
hypothetical protein, partial [Acinetobacter baumannii]	38.0	0.093	12/15(80%)	13/15(86%)	0/15(0%)
Core histone H2A/H2B/H3/H4 [Chlamydia trachomatis]	37.1	0.18	12/15(80%)	13/15(86%)	0/15(0%)
site-specific integrase [Fusobacterium nucleatum]	34.6	1.4	10/13(77%)	11/13(84%)	0/13(0%)
hypothetical protein [Curvibacter sp. AEP1-3]	31.2	23	9/10(90%)	10/10(100%)	0/10(0%)
hypothetical protein [Nitrosomonas sp. Nm134]	31.2	23	9/10(90%)	10/10(100%)	0/10(0%)

**Suppl. Table 2B.** CENPA sequence “LQEAAEAFLVHLFED” BLASTp matches from viruses.

**Description**	**Score**	**E value**	**Identities**	**Positives**	**Gaps**
hypothetical protein DSLPV1_013 [Dishui lake phycodnavirus 1]	37.5	0.002	12/15(80%)	13/15(86%)	0/15(0%)
histone H3 [Sylvanvirus sp.]	30.8	0.51	10/15(67%)	11/15(73%)	0/15(0%)
putative DNA polymerase [Agrobacterium phage 7-7-1]	27.8	5.8	8/8(100%)	8/8(100%)	0/8(0%)
hypothetical protein [Yersinia phage fPS-59]	26.5	16	8/9(89%)	8/9(88%)	0/9(0%)
HNS binding protein [Yersinia phage fPS-89]	26.5	16	8/9(89%)	8/9(88%)	0/9(0%)
hypothetical protein pmac_cds_685 [Pandoravirus macleodensis]	26.1	24	7/8(88%)	7/8(87%)	0/8(0%)
envelope glycoprotein gp120, partial [Human immunodeficiency virus 1]	25.7	33	9/14(64%)	9/14(64%)	5/14(35%)
hypothetical protein [Roseovarius Plymouth podovirus 1]	25.7	33	8/11(73%)	10/11(90%)	0/11(0%)
hypothetical protein [Roseovarius sp. 217 phage 1]	25.7	33	8/11(73%)	10/11(90%)	0/11(0%)
virion protein US2 [Meleagrid alphaherpesvirus 1]	25.7	33	7/10(70%)	8/10(80%)	0/10(0%)
hypothetical protein PAN70_054 [Pseudomonas phage PAN70]	25.7	33	7/10(70%)	9/10(90%)	0/10(0%)
hypothetical protein [Aeromonas phage 4_L372XY]	25.2	46	8/14(57%)	10/14(71%)	1/14(7%)
hypothetical protein [Erwinia phage phiEaH2]	24.8	66	7/8(88%)	7/8(87%)	0/8(0%)
ypothetical protein ASESINO_96 [Erwinia phage vB_EamM_Asesino]	24.8	66	7/8(88%)	7/8(87%)	0/8(0%)
RNA-dependent RNA polymerase, partial [Turkey picobirnavirus]	24.8	67	9/15(60%)	10/15(66%)	0/15(0%)
RNA-dependent RNA polymerase, partial [Turkey picobirnavirus]	24.8	67	9/15(60%)	10/15(66%)	0/15(0%)
Putative DNA primase [Prokaryotic dsDNA virus sp.]	24.8	67	9/15(60%)	9/15(60%)	6/15(40%)
encapsidation protein 52K [Bat mastadenovirus]	24.8	67	7/7(100%)	7/7(100%)	0/7(0%)
putative DNA polymerase [Prokaryotic dsDNA virus sp.]	24.8	67	7/8(88%)	8/8(100%)	0/8(0%)
hypothetical protein HUXLEY_63 [Erwinia phage vB_EamM_Huxley]	24.4	94	7/8(88%)	8/8(100%)	0/8(0%)
hypothetical protein Tp1109DCM542121_60 [Prokaryotic dsDNA virus sp.]	24.4	95	7/8(88%)	7/8(87%)	0/8(0%)
hypothetical protein GOVbin2277_77 [Prokaryotic dsDNA virus sp.]	24.0	134	7/7(100%)	7/7(100%)	0/7(0%)
HNH endonuclease [Arthrobacter phage Hestia]	24.0	134	7/7(100%)	7/7(100%)	0/7(0%)
hypothetical protein [Yersinia phage fEV-1]	24.0	134	7/7(100%)	7/7(100%)	0/7(0%)
replication associated protein [Cotton leaf curl Multan alphasatellite]	4.0	135	6/7(86%)	7/7(100%)	0/7(0%)
hypothetical protein phiLo_113 [Thermus phage phiLo]	24.0	135	6/7(86%)	7/7(100%)	0/7(0%)
tapemeasure protein [Gordonia phage Toniann]	24.0	136	7/7(100%)	7/7(100%)	0/7(0%)
ape measure protein [Gordonia phage Cucurbita]	24.0	136	7/7(100%)	7/7(100%)	0/7(0%)
tapemeasure protein [Gordonia phage ClubL]	24.0	136	7/7(100%)	7/7(100%)	0/7(0%)
tape measure protein [Gordonia phage Aphelion]	24.0	136	7/7(100%)	7/7(100%)	0/7(0%)
tape measure protein [Gordonia phage Smoothie]	24.0	136	7/7(100%)	7/7(100%)	0/7(0%)
tape measure protein [Gordonia phage WilliamBoone]	24.0	136	7/7(100%)	7/7(100%)	0/7(0%)
tapemeasure protein [Gordonia phage Bachita]	24.0	136	7/7(100%)	7/7(100%)	0/7(0%)
WSSV530 [Shrimp white spot syndrome virus]	23.5	189	7/8(88%)	7/8(87%)	0/8(0%)
wsv471 [Shrimp white spot syndrome virus]	23.5	189	7/8(88%)	7/8(87%)	0/8(0%)
P13 [Hamiltonella virus APSE1]	23.5	190	7/8(88%)	7/8(87%)	0/8(0%)
lysozyme [Bacteriophage APSE-4]	23.5	190	7/8(88%)	7/8(87%)	0/8(0%)
hypothetical protein SEA_PARADIDDLES_142 [Streptomyces phage Paradiddles]	23.5	190	6/6(100%)	6/6(100%)	0/6(0%)
ypothetical protein SEA_PEEBS_144 [Streptomyces phage Peebs]	23.5	190	6/6(100%)	6/6(100%)	0/6(0%)
hypothetical protein 162285194 [Organic Lake phycodnavirus 1]	23.5	190	6/6(100%)	6/6(100%)	0/6(0%)
RNA-dependent RNA polymerase, partial [Porcine picobirnavirus]	23.5	190	7/9(78%)	8/9(88%)	0/9(0%)
hypothetical protein SEA_MILDRED21_153 [Streptomyces phage Mildred21]	23.5	190	6/6(100%)	6/6(100%)	0/6(0%)
peptidase [Streptomyces phage Braelyn]	23.5	190	6/6(100%)	6/6(100%)	0/6(0%)
hypothetical protein SEA_NOOTNOOT_143 [Streptomyces phage NootNoot]	3.5	190	6/6(100%)	6/6(100%)	0/6(0%)
peptidase [Streptomyces phage Teutsch]	3.5	190	6/6(100%)	6/6(100%)	0/6(0%)
hypothetical protein SEA_SUSHI23_147 [Streptomyces phage Sushi23]	3.5	190	6/6(100%)	6/6(100%)	0/6(0%)
peptidase [Streptomyces phage Starbow]	3.5	190	6/6(100%)	6/6(100%)	0/6(0%)
peptidase [Streptomyces phage Daubenski]	3.5	190	6/6(100%)	6/6(100%)	0/6(0%)
peptidase [Streptomyces phage Karimac]	3.5	190	6/6(100%)	6/6(100%)	0/6(0%)
peptidase [Streptomyces phage Tribute]	3.5	190	6/6(100%)	6/6(100%)	0/6(0%)
peptidase [Streptomyces phage EGole]	3.5	190	6/6(100%)	6/6(100%)	0/6(0%)
peptidase [Streptomyces phage Evy]	3.5	190	6/6(100%)	6/6(100%)	0/6(0%)
peptidase [Streptomyces phage Wipeout]	3.5	190	6/6(100%)	6/6(100%)	0/6(0%)
peptidase [Streptomyces phage LukeCage]	3.5	190	6/6(100%)	6/6(100%)	0/6(0%)
hypothetical protein SEA_YABOI_154 [Streptomyces phage Yaboi]	3.5	190	6/6(100%)	6/6(100%)	0/6(0%)
peptidase [Streptomyces phage Wofford]	3.5	190	6/6(100%)	6/6(100%)	0/6(0%)
peptidase [Streptomyces phage StarPlatinum]	3.5	190	6/6(100%)	6/6(100%)	0/6(0%)
hypothetical protein PBI_JAY2JAY_152 [Streptomyces phage Jay2Jay]	23.5	191	6/6(100%)	6/6(100%)	0/6(0%)
hypothetical protein SEA_WARPY_151 [Streptomyces phage Warpy]	23.5	191	6/6(100%)	6/6(100%)	0/6(0%)
repeat element protein-c18.1 [Hyposoter fugitivus ichnovirus]	23.5	191	6/6(100%)	6/6(100%)	0/6(0%)
integrase family protein [uncultured Mediterranean phage]	23.5	192	7/10(70%)	9/10(90%)	0/10(0%)
hypothetical protein YASMINEVIRUS_1387 [Yasminevirus sp. GU-2018]	23.5	192	8/9(89%)	8/9(88%)	1/9(11%)
large T antigen, partial [Tasmanian devil-associated polyoma-like virus 2	23.5	192	8/12(67%)	10/12(83%)	1/12(8%)

**Suppl. Table 2C.** CENPA sequence “LQEAAEAFLVHLFED” BLASTp matches from eukaryotes.

**Description**	**Score**	**E value**	**Identities**	**Positives**	**Gaps**
histone H3-like centromeric protein hH3v [Emmonsia sp. CAC-2015a]	51.1	6.00E-07	100.00%	15/15(100%)	0/15(0%)
hypothetical protein AN6554.2 [Aspergillus nidulans FGSC A4]	51.1	6.00E-07	100.00%	15/15(100%)	0/15(0%)
hypothetical protein V501_04928 [Pseudogymnoascus sp. VKM F-4519 (FW-2642)]	51.1	7.00E-07	100.00%	15/15(100%)	0/15(0%)
hypothetical protein AC578_6719 [Pseudocercospora eumusae]	51.1	7.00E-07	100.00%	15/15(100%)	0/15(0%)
hypothetical protein AC579_7423 [Pseudocercospora musae]	51.1	7.00E-07	100.00%	15/15(100%)	0/15(0%)
94d01dbe-7812-426c-b7ef-62d6d5cc3996 [Thermothielavioides terrestris]	51.1	7.00E-07	100.00%	15/15(100%)	0/15(0%)
hypothetical protein AUEXF2481DRAFT_64859 [Aureobasidium subglaciale EXF-2481]	51.1	7.00E-07	100.00%	15/15(100%)	0/15(0%)
centromeric DNA-binding histone H3-like protein cse4 [Pseudogymnoascus destructans]	51.1	7.00E-07	100.00%	15/15(100%)	0/15(0%)
uncharacterized protein THITE_113981 [Thermothielavioides terrestris NRRL 8126]	51.1	7.00E-07	100.00%	15/15(100%)	0/15(0%)
histone H3 [Kwoniella heveanensis CBS 569]	51.1	7.00E-07	100.00%	15/15(100%)	0/15(0%)
histone H3 [Kwoniella heveanensis BCC8398]	51.1	7.00E-07	100.00%	15/15(100%)	0/15(0%)
Similar to Histone H3-like centromeric protein cnp1; acc. no. Q9Y812 [Pyronema omphalodes CBS 100304]	51.1	7.00E-07	100.00%	15/15(100%)	0/15(0%)
histone H3-like centromeric protein cnp1 [Smittium mucronatum]	51.1	7.00E-07	100.00%	15/15(100%)	0/15(0%)
centromere protein Cse4, putative [Talaromyces marneffei ATCC 18224]	51.1	7.00E-07	100.00%	15/15(100%)	0/15(0%)
hypothetical protein BHQ10_008142 [Talaromyces amestolkiae]	51.1	7.00E-07	100.00%	15/15(100%)	0/15(0%)
Histone-fold [Penicillium sp. ‘occitanis’]	51.1	7.00E-07	100.00%	15/15(100%)	0/15(0%)
centromere protein [Talaromyces cellulolyticus]	51.1	7.00E-07	100.00%	15/15(100%)	0/15(0%)
hypothetical protein ZTR_08717 [Talaromyces verruculosus]	51.1	7.00E-07	100.00%	15/15(100%)	0/15(0%)
Histone 3-like protein [Rasamsonia emersonii CBS 393.64]	51.1	7.00E-07	100.00%	15/15(100%)	0/15(0%)
centromere protein Cse4, putative [Talaromyces stipitatus ATCC 10500]	51.1	7.00E-07	100.00%	15/15(100%)	0/15(0%)
hypothetical protein ASPACDRAFT_46181 [Aspergillus aculeatus ATCC 16872]	51.1	7.00E-07	100.00%	15/15(100%)	0/15(0%)
histone-fold-containing protein [Aspergillus fijiensis CBS 313.89]	51.1	7.00E-07	100.00%	15/15(100%)	0/15(0%)
Uncharacterized protein DSM5745_04281 [Aspergillus mulundensis]	51.1	7.00E-07	100.00%	15/15(100%)	0/15(0%)
putative centromere protein Cse4 [Aspergillus novofumigatus IBT 16806]	51.1	7.00E-07	100.00%	15/15(100%)	0/15(0%)
histone-fold-containing protein [Aspergillus violaceofuscus CBS 115571]	51.1	7.00E-07	100.00%	15/15(100%)	0/15(0%)
histone-fold-containing protein [Aspergillus japonicus CBS 114.51]	51.1	7.00E-07	100.00%	15/15(100%)	0/15(0%)
hypothetical protein Egran_04734 [Elaphomyces granulatus]	51.1	7.00E-07	100.00%	15/15(100%)	0/15(0%)
Histone H3-like centromeric protein cse-4 [Talaromyces islandicus]	51.1	7.00E-07	100.00%	15/15(100%)	0/15(0%)
putative centromere protein Cse4 [Aspergillus brunneoviolaceus CBS 621.78]	51.1	7.00E-07	100.00%	15/15(100%)	0/15(0%)
histone H3-like centromeric protein A [Bos indicus × Bos taurus]	51.1	7.00E-07	100.00%	15/15(100%)	0/15(0%)
putative centromere protein Cse4 [Aspergillus uvarum CBS 121591]	51.1	7.00E-07	100.00%	15/15(100%)	0/15(0%)
centromeric DNA-binding histone H3-like protein cse4 [Monascus purpureus]	51.1	7.00E-07	100.00%	15/15(100%)	0/15(0%)
hypothetical protein ASPGLDRAFT_49919 [Aspergillus glaucus CBS 516.65]	51.1	7.00E-07	100.00%	15/15(100%)	0/15(0%)
TPA: Histone H3-like centromeric protein A-like [Bos taurus]	51.1	7.00E-07	100.00%	15/15(100%)	0/15(0%)
TPA: hypothetical protein similar to histone H3 (Broad) [Aspergillus nidulans FGSC A4]	51.1	7.00E-07	100.00%	15/15(100%)	0/15(0%)
PREDICTED: histone H3-like centromeric protein A [Bison bison bison]	51.1	7.00E-07	100.00%	15/15(100%)	0/15(0%)
histone-fold-containing protein [Aspergillus indologenus CBS 114.80]	51.1	7.00E-07	100.00%	15/15(100%)	0/15(0%)
putative centromere protein Cse4 [Aspergillus aculeatinus CBS 121060]	51.1	7.00E-07	100.00%	15/15(100%)	0/15(0%)
histone H3 variant [Aspergillus ruber CBS 135680]	51.1	7.00E-07	100.00%	15/15(100%)	0/15(0%)
hypothetical protein ASPZODRAFT_135809 [Penicilliopsis zonata CBS 506.65]	51.1	7.00E-07	100.00%	15/15(100%)	0/15(0%)
hypothetical protein SI65_03462 [Aspergillus cristatus]	51.1	7.00E-07	100.00%	15/15(100%)	0/15(0%)
histone H3-like centromeric protein cse-4 [Helicocarpus griseus UAMH5409]	51.1	7.00E-07	100.00%	15/15(100%)	0/15(0%)
histone H3 [Kwoniella mangroviensis CBS 8507]	51.1	7.00E-07	100.00%	15/15(100%)	0/15(0%)
histone H3 variant [Aspergillus vadensis CBS 113365]	51.1	7.00E-07	100.00%	15/15(100%)	0/15(0%)
histone H3 variant [Aspergillus kawachii IFO 4308]	51.1	7.00E-07	100.00%	15/15(100%)	0/15(0%)
histone-fold-containing protein [Aspergillus taichungensis]	51.1	7.00E-07	100.00%	15/15(100%)	0/15(0%)
histone H3-like centromeric protein A [Mus pahari]	51.1	7.00E-07	100.00%	15/15(100%)	0/15(0%)
histone H3 variant [Aspergillus neoniger CBS 115656]	51.1	7.00E-07	100.00%	15/15(100%)	0/15(0%)
histone H3-like centromeric protein CSE4 [Kwoniella bestiolae CBS 10118]	51.1	7.00E-07	100.00%	15/15(100%)	0/15(0%)
histone H3-like centromeric protein hH3v [Blastomyces percursus]	51.1	7.00E-07	100.00%	15/15(100%)	0/15(0%)
hypothetical protein ASPBRDRAFT_132387 [Aspergillus brasiliensis CBS 101740]	51.1	7.00E-07	100.00%	15/15(100%)	0/15(0%)
histone H3 variant [Aspergillus eucalypticola CBS 122712]	51.1	7.00E-07	100.00%	15/15(100%)	0/15(0%)
histone H3-like centromeric protein A [Blastomyces silverae]	51.1	7.00E-07	100.00%	15/15(100%)	0/15(0%)
histone H3-like centromeric protein cse-4 [Aspergillus niger CBS 513.88]	51.1	7.00E-07	100.00%	15/15(100%)	0/15(0%)
histone-fold-containing protein [Aspergillus novoparasiticus]	51.1	7.00E-07	100.00%	15/15(100%)	0/15(0%)
histone H3-like centromeric protein cse-4 [Aspergillus bombycis]	51.1	7.00E-07	100.00%	15/15(100%)	0/15(0%)
hypothetical protein BDV24DRAFT_136081 [Aspergillus arachidicola]	51.1	7.00E-07	100.00%	15/15(100%)	0/15(0%)
histone-fold-containing protein [Aspergillus bertholletius]	51.1	7.00E-07	100.00%	15/15(100%)	0/15(0%)
histone H3-like centromeric protein cse-4 [Aspergillus nomius NRRL 13137]	51.1	7.00E-07	100.00%	15/15(100%)	0/15(0%)
histone-fold-containing protein [Aspergillus parasiticus]	51.1	7.00E-07	100.00%	15/15(100%)	0/15(0%)
histone H3 [Aspergillus sclerotialis]	51.1	7.00E-07	100.00%	15/15(100%)	0/15(0%)
histone-fold-containing protein [Aspergillus sergii]	51.1	7.00E-07	100.00%	15/15(100%)	0/15(0%)
histone-fold-containing protein [Aspergillus transmontanensis]	51.1	7.00E-07	100.00%	15/15(100%)	0/15(0%)
histone-fold-containing protein [Aspergillus pseudotamarii]	51.1	7.00E-07	100.00%	15/15(100%)	0/15(0%)
histone-fold-containing protein [Aspergillus pseudocaelatus]	51.1	7.00E-07	100.00%	15/15(100%)	0/15(0%)
histone-fold-containing protein [Aspergillus alliaceus]	51.1	7.00E-07	100.00%	15/15(100%)	0/15(0%)
histone-fold-containing protein [Cenococcum geophilum 1.58]	51.1	7.00E-07	100.00%	15/15(100%)	0/15(0%)
histone-fold-containing protein [Aspergillus candidus]	51.1	7.00E-07	100.00%	15/15(100%)	0/15(0%)
histone-fold-containing protein [Aspergillus caelatus]	51.1	7.00E-07	100.00%	15/15(100%)	0/15(0%)
histone-fold-containing protein [Aspergillus nomius]	51.1	7.00E-07	100.00%	15/15(100%)	0/15(0%)
centromere protein [Aspergillus flavus AF70]	51.1	7.00E-07	100.00%	15/15(100%)	0/15(0%)
putative centromere protein Cse4 [Aspergillus homomorphus CBS 101889]	51.1	7.00E-07	100.00%	15/15(100%)	0/15(0%)
histone H3-like centromeric protein cse-4 [Aspergillus ibericus CBS 121593]	51.1	7.00E-07	100.00%	15/15(100%)	0/15(0%)
histone H3 variant [Aspergillus sclerotioniger CBS 115572]	51.1	7.00E-07	100.00%	15/15(100%)	0/15(0%)
histone H3-like centromeric protein cse-4 [Aspergillus sclerotiicarbonarius CBS 121057]	51.1	7.00E-07	100.00%	15/15(100%)	0/15(0%)
histone H3 variant [Aspergillus saccharolyticus JOP 1030-1]	51.1	7.00E-07	100.00%	15/15(100%)	0/15(0%)
histone H3 [Histoplasma capsulatum NAm1]	51.1	7.00E-07	100.00%	15/15(100%)	0/15(0%)
histone H3-like centromeric protein A [Blastomyces gilchristii SLH14081]	51.1	7.00E-07	100.00%	15/15(100%)	0/15(0%)
hypothetical protein ASPCADRAFT_177696 [Aspergillus carbonarius ITEM 5010]	51.1	7.00E-07	100.00%	15/15(100%)	0/15(0%)
histone H3 variant, putative [Aspergillus fischeri NRRL 181]	51.1	7.00E-07	100.00%	15/15(100%)	0/15(0%)
centromere protein Cse4, putative [Aspergillus fumigatus Af293]	51.1	7.00E-07	100.00%	15/15(100%)	0/15(0%)
histone H3-like centromeric protein hH3v [Emmonsia crescens]	51.1	7.00E-07	100.00%	15/15(100%)	0/15(0%)
histone H3-like centromeric protein hH3v [Aspergillus lentulus]	51.1	7.00E-07	100.00%	15/15(100%)	0/15(0%)
centromere protein Cse4 [Aspergillus fumigatus Z5]	51.1	7.00E-07	100.00%	15/15(100%)	0/15(0%)
centromere protein Cse4 [Aspergillus fumigatus var. RP-2014]	51.1	7.00E-07	100.00%	15/15(100%)	0/15(0%)
histone-fold-containing protein [Aspergillus leporis]	51.1	7.00E-07	100.00%	15/15(100%)	0/15(0%)
histone H3-like centromeric protein A [Emmonsia crescens UAMH 3008]	51.1	7.00E-07	100.00%	15/15(100%)	0/15(0%)
histone H3 [Byssochlamys spectabilis]	51.1	7.00E-07	100.00%	15/15(100%)	0/15(0%)
histone-fold-containing protein [Aspergillus tamarii]	51.1	7.00E-07	100.00%	15/15(100%)	0/15(0%)
histone H3-like centromeric protein cse-4 [Aspergillus udagawae]	51.1	7.00E-07	100.00%	15/15(100%)	0/15(0%)
histone H3 [Aspergillus terreus NIH2624]	51.1	7.00E-07	100.00%	15/15(100%)	0/15(0%)
centromeric DNA-binding histone H3-like protein cse4 [Aspergillus tanneri]	51.1	7.00E-07	100.00%	15/15(100%)	0/15(0%)
histone H3-like centromeric protein cnp1 [Blastomyces parvus]	51.1	7.00E-07	100.00%	15/15(100%)	0/15(0%)
histone H3-like centromeric protein hH3v [Aspergillus thermomutatus]	51.1	7.00E-07	100.00%	15/15(100%)	0/15(0%)
hypothetical protein ATETN484_0008013300 [Aspergillus terreus]	51.1	7.00E-07	100.00%	15/15(100%)	0/15(0%)
histone H3 [Aspergillus steynii IBT 23096]	51.1	7.00E-07	100.00%	15/15(100%)	0/15(0%)
histone H3-like centromeric protein hH3v [Aspergillus turcosus]	51.1	7.00E-07	100.00%	15/15(100%)	0/15(0%)
histone H3 [Aspergillus avenaceus]	51.1	7.00E-07	100.00%	15/15(100%)	0/15(0%)
histone H3-like centromeric protein CSE4 [Paracoccidioides brasiliensis Pb18]	51.1	7.00E-07	100.00%	15/15(100%)	0/15(0%)
histone H3 [Paracoccidioides lutzii Pb01]	51.1	7.00E-07	100.00%	15/15(100%)	0/15(0%)

**Suppl. Table 2D.** CENPA sequence “LQEAAEAFLVHLFED” BLASTp matches from Gram+ bacteria, in particular.

**Description**	**Score**	**E value**	**Identities**	**Positives**	**Gaps**
hypothetical protein, partial [Macrococcus caseolyticus]	41.8 bits(91)	6.00E-04	13/15(87%)	14/15(93%)	0/15(0%)
hypothetical protein D6861_11520 [Macrococcus caseolyticus]	41.8 bits(91)	6.00E-04	13/15(87%)	14/15(93%)	0/15(0%)
hypothetical protein DKP78_14540, partial [Enterococcus faecium]	40.5 bits(88)	0.002	13/15(87%)	14/15(93%)	0/15(0%)
histone H3, partial [Soehngenia saccharolytica]	40.5 bits(88)	0.002	13/15(87%)	14/15(93%)	0/15(0%)
histone H3 [Paenibacillus sp. IHB B 3415]	40.5 bits(88)	0.002	13/15(87%)	14/15(93%)	0/15(0%)
histone H3 [Bacillus paralicheniformis]	40.5 bits(88)	0.002	13/15(87%)	14/15(93%)	0/15(0%)
hypothetical protein QW71_36435 [Paenibacillus sp. IHB B 3415]	40.5 bits(88)	0.002	13/15(87%)	14/15(93%)	0/15(0%)
histone H3 [Paenibacillus azotifigens]	38.8 bits(84)	0.007	12/15(80%)	13/15(86%)	
hypothetical protein E8P77_31285, partial [Soehngenia saccharolytica]	38.0 bits(82)	0.015	12/15(80%)	13/15(86%)	0/15(0%)
hypothetical protein, partial [Enterococcus faecium]	38.0 bits(82)	0.015	12/15(80%)	13/15(86%)	

**Suppl. Table 2E.** CENPA sequence “LQEAAEAFLVHLFED” BLASTp matches from enterococci, in particular.

**Description**	**Score**	**E value**	**Identities**	**Positives**	**Gaps**
hypothetical protein DKP78_14540, partial [Enterococcus faecium]	40.5 bits(88)	6.00E-05	13/15(87%)	14/15(93%)	0/15(0%)
hypothetical protein, partial [Enterococcus faecium]	38.0 bits(82)	5.00E-04	12/15(80%)	13/15(86%)	0/15(0%)
ABC transporter permease/substrate-binding protein [Enterococcus sulfureus]	27.8 bits(58)	2.0	8/8(100%)	8/8(100%)	0/8(0%)
HAD-IA family hydrolase, partial [Enterococcus faecalis]	26.9 bits(56)	4.0	8/11(73%)	8/11(72%)	0/11(0%)
MULTISPECIES: HAD family hydrolase [Bacilli]	26.9 bits(56)	4.0	8/11(73%)	8/11(72%)	0/11(0%)
HAD family hydrolase [Enterococcus faecalis]	26.9 bits(56)	4.0	8/11(73%)	8/11(72%)	0/11(0%)
HAD family hydrolase [Enterococcus faecalis]	26.9 bits(56)	4.0	8/11(73%)	8/11(72%)	0/11(0%)
HAD hydrolase, family IA, variant 1 [Enterococcus faecalis TX0411]	26.9 bits(56)	4.0	8/11(73%)	8/11(72%)	0/11(0%)
Phosphoglycolate phosphatase [Enterococcus faecalis GA2]	26.9 bits(56)	4.0	8/11(73%)	8/11(72%)	0/11(0%)
HAD hydrolase, family IA, variant 1 [Enterococcus faecalis EnGen0297]	26.9 bits(56)	4.0	8/11(73%)	8/11(72%)	0/11(0%)
HAD family hydrolase [Enterococcus faecalis]	26.9 bits(56)	4.0	8/11(73%)	8/11(72%)	0/11(0%)
phosphopantothenate--cysteine ligase [Enterococcus mundtii]	25.7 bits(53)	11	8/10(80%)	8/10(80%)	0/11(0%)
phosphopantothenate--cysteine ligase [Enterococcus mundtii]	25.7 bits(53)	11	8/10(80%)	8/10(80%)	0/11(0%)
phosphopantothenate--cysteine ligase [Enterococcus mundtii]	25.7 bits(53)	11	8/10(80%)	8/10(80%)	0/11(0%)
hypothetical protein [Enterococcus faecium]	25.7 bits(53)	11	8/10(80%)	8/10(80%)	0/11(0%)
hypothetical protein A5852_003495 [Enterococcus faecium]	25.7 bits(53)	11	8/10(80%)	8/10(80%)	0/11(0%)

**Suppl. Table 2F.** CENPA sequence “LQEAAEAFLVHLFED” BLASTp matches from streptococci, in particular.

**Description**	**Score**	**E value**	**Identities**	**Positives**	**Gaps**
adenine-specific methyltransferase [Streptococcus pneumoniae]	27.8	6.5	9/12(75%)	9/12(75%)	3/12(25%)
IS256 family transposase [Streptococcus thermophilus]	27.8	6.5	10/14(71%)	11/14(78%)	0/14(0%)
IS256 family transposase [Streptococcus thermophilus]	27.8	6.5	10/14(71%)	11/14(78%)	0/14(0%)
IS256 family transposase [Streptococcus thermophilus]	27.8	6.5	10/14(71%)	11/14(78%)	0/14(0%)
IS1191, transposase, IS256 family [Streptococcus thermophilus LMG 18311]	27.8	6.5	10/14(71%)	11/14(78%)	0/14(0%)
hydrolase [Streptococcus agalactiae]	26.9	13	8/11(73%)	8/11(72%)	0/11(0%)
methyltransferase [Streptococcus massiliensis]	26.9	13	8/9(89%)	8/9(88%)	0/9(0%)
class I SAM-dependent rRNA methyltransferase [Streptococcus massiliensis]	26.9	13	8/9(89%)	8/9(88%)	0/9(0%)
GNAT family N-acetyltransferase [Streptococcus sp. DD12]	26.5	19	9/12(75%)	10/12(83%)	0/12(0%)
hypothetical protein, partial [Streptococcus suis]	26.5	19	7/7(100%)	7/7(100%)	0/7(0%)
hypothetical protein, partial [Streptococcus suis]	26.5	19	7/7(100%)	7/7(100%)	0/7(0%)
hypothetical protein, partial [Streptococcus suis]	26.5	19	7/7(100%)	7/7(100%)	0/7(0%)
hypothetical protein, partial [Streptococcus suis]	26.5	19	7/7(100%)	7/7(100%)	0/7(0%)
hypothetical protein, partial [Streptococcus suis]	26.5	19	7/7(100%)	7/7(100%)	0/7(0%)
zinc metalloprotease ZmpC [Streptococcus suis]	26.5	19	7/7(100%)	7/7(100%)	0/7(0%)
hypothetical protein, partial [Streptococcus suis]	26.5	19	7/7(100%)	7/7(100%)	0/7(0%)
hypothetical protein, partial [Streptococcus suis]	26.5	19	7/7(100%)	7/7(100%)	0/7(0%)
hypothetical protein XK27_08180 [Streptococcus suis]	26.5	19	7/7(100%)	7/7(100%)	0/7(0%)
hypothetical protein, partial [Streptococcus suis]	26.5	19	7/7(100%)	7/7(100%)	0/7(0%)
hypothetical protein, partial [Streptococcus suis]	26.5	19	7/7(100%)	7/7(100%)	0/7(0%)

**Suppl. Table 2G.** CENPA sequence “LQEAAEAFLVHLFED” BLASTp matches from *Helicobacter* species, in particular.

**Description**	**Score**	**E value**	**Identities**	**Positives**	**Gaps**
nickel-dependent hydrogenase large subunit [Helicobacter aurati]	29.1	1.2	11/19(58%)	11/19(57%)	7/19(36%)
nickel-dependent hydrogenase large subunit [Helicobacter saguini]	27.8	3.3	11/19(58%)	12/19(63%)	7/19(36%)
hypothetical protein [Helicobacter jaachi]	26.1	13	7/8(88%)	7/8(87%)	0/8(0%)
nickel-dependent hydrogenase large subunit [Helicobacter sp. MIT 14-3879]	26.1	13	10/19(53%)	10/19(52%)	7/19(36%)
16S rRNA (guanine(966)-N(2))-methyltransferase RsmD [Helicobacter sp.]	25.7	19	7/9(78%)	7/9(77%)	0/9(0%)
uracil-DNA glycosylase [Helicobacter pylori]	25.2	27	8/13(62%)	9/13(69%)	4/13(30%)

**Suppl. Table 3.** Taxid ID numbers used for BLASTp search analysis.

**Organism**	**Taxid ID Number**
Human	9606
Eukaryotes	2759

Bacteria	2
Gram positive bacteria	1239
Enterococcus	1350
Streptococcus	1301
Helicobacter	209
Brucella	234
Staphylococcus	1279

Viruses	10239
Herpesviridiae	10292
Epstein-Barr virus	10376
Human cytomegalovirus	10359
Hepatitis C virus	11103

## ABBREVIATIONS:

aa: Amino acid
Ab: Antibody
AutoAb: Autoantibody
ARD: Autoimmune rheumatic diseases
SjS: Sjögren’s syndrome
SLE: Systemic lupus erythematosus
SSc: Systemic sclerosis
